# *G6PD Potenza*: A Novel Pathogenic Variant Broadening the Mutational Landscape in the Italian Population

**DOI:** 10.3390/genes15101298

**Published:** 2024-10-04

**Authors:** Claudio Ricciardi Tenore, Eugenia Tulli, Claudia Calò, Roberto Bertozzi, Jessica Evangelista, Giulia Maneri, Martina Rinelli, Francesca Brisighelli, Alessia Perrucci, Elisa De Paolis, Andrea Urbani, Maria De Bonis, Angelo Minucci

**Affiliations:** 1 Departmental Unit of Molecular and Genomic Diagnostics, Fondazione Policlinico Universitario Agostino Gemelli IRCCS, 00168 Rome, Italy; claudio.ricciarditenore@guest.policlinicogemelli.it (C.R.T.); eugenia.tulli01@icatt.it (E.T.); claudia.calo@policlinicogemelli.it (C.C.); roberto.bertozzi01@icatt.it (R.B.); jessica.evangelista@policlinicogemelli.it (J.E.); giulia.maneri@guest.policlinicogemelli.it (G.M.); martina.rinelli@guest.policlinicogemelli.it (M.R.); francesca.brisghelli@policlinicogemelli.it (F.B.); alessia.perrucci01@icatt.it (A.P.); elisa.depaolis@policlinicogemelli.it (E.D.P.); maria.debonis@policlinicogemelli.it (M.D.B.); 2 Genomics Research Core Facility, Gemelli Science and Technology Park (GSTeP), Fondazione Policlinico Universitario Agostino Gemelli IRCCS, 00168 Rome, Italy; 3 Department of Basic Biotechnological Sciences, Intensivological and Perioperative Clinics, Università Cattolica del Sacro Cuore, 00168 Rome, Italy; 4 Departmental Unity of Chemistry, Biochemistry and Clinical Molecular Biology, Department of Diagnostic and Laboratory Medicine, Fondazione Policlinico Universitario Agostino Gemelli IRCCS, 00168 Rome, Italy

**Keywords:** acute haemolytic anaemia, Potenza, Sanger sequencing, *G6PD* deficiency

## Abstract

**Background:** Glucose 6 phosphate dehydrogenase (*G6PD)* is a rate-limiting enzyme of the pentose phosphate pathway. The loss of *G6PD* activity in red blood cells increases the risk of acute haemolytic anaemia under oxidative stress induced by infections, some medications, or fava beans. More than 200 single missense mutations are known in the *G6PD* gene. A 41-year-old woman with a family history of favism coming from the Basilicata region (Italy) was evaluated at our hospital for *G6PD* abnormalities. **Methods:** DNA was extracted from a peripheral blood sample and genotyped for the most common *G6PD* pathogenic variants (PVs). Positive results obtained by Restriction Fragment Length Polymorphism (*RFLP*), as per practice in our laboratory, were then reconfirmed in Sanger sequencing. **Results:** *RFLP* analysis highlighted a variant compatible with the *G6PD Cassano* variant. Confirmatory testing by Sanger unexpectedly identified a novel variant: c.1357G>A, p.(Val453Met) (NM_001360016.2); the same variant was found in the patient’s mother. In silico models predicted a deleterious effect of this variant at the protein level. The novel *G6PD* variant was named “*G6PD Potenza*” on the basis of the patient’s regional origin. **Conclusions:** This case describes a novel *G6PD* variant. It also highlights how the Sanger sequencing technique still represents an indispensable confirmatory standard method for variants that could be misinterpreted by only using a “first-level” approach, such as the *RFLP*. We stress that the evaluation of clinical manifestations in G6PD-deficient patients is of primary importance for the classification of each new *G6PD* mutation, in agreement with the new WHO guidelines.

## 1. Introduction

Glucose-6-phosphate dehydrogenase (*G6PD*) is a cytosolic rate-limiting enzyme of the pentose phosphate pathway (*PPP*) that breaks down glucose, promotes the oxidation of β-D-glucose-6-phosphate to D-glucono-1,5-lactone-6-phosphate, and produces a reduced form of nicotinamide adenine dinucleotide phosphate (*NADPH*) as a byproduct in the oxidative phase. Both enzymatic and genetic tests allow for the evaluation of *G6PD* status. *G6PD* deficiency affects nearly 400 million people in the world and it is more commonly observed in males compared to females [1]. *G6PD* deficiency occurs most frequently in Asia, Africa, the Mediterranean, and the Middle East, synchronizing with endemic malaria [2]. In Italy, *G6PD* deficiency is mainly present in Sardinia and Sicily (2–15%), but can also be found in the Campania, Basilicata, Puglia, and Lazio regions [3]. The spread of these variants is closely correlated with malaria: in areas where the disease was endemic, the *G6PD Mediterranean*, *G6PD Cassano*, *G6PD Seattle,* and *G6PD A*-pathogenic variants (PVs) are mainly observed [4].

More than 200 *G6PD* PVs are known [5], and recently, by searching publicly available databases, 1041 variants have been listed [6]. The World Health Organization (*WHO*) has recently revised their classification [7]: variants in class A (formerly class I) are the more severe group, as they are associated with life-long chronic non-spherocytic haemolytic anaemia (CNSHA); persons with variants in class B (formerly class II and class III) are asymptomatic in the steady state, but they are at risk of acute haemolytic anaemia (AHA) that may be triggered by eating fava beans, by infections, or by certain drugs [8,9].

In this study, we reported the clinical and genetic data of an asymptomatic 41-year-old woman that underwent *G6PD* gene testing, resulting in the presence of an unreported variant (c.1357G>A, NM_001360016.2). The combined use of Restriction Fragment Length Polymorphism (*RFLP*) and Sanger sequencing as a confirmatory method allowed for the correct annotation of the variant, initially misinterpreted as the *G6PD Cassano* variant [10].

It was decided to name the novel variant “*G6PD Potenza*”, with regard to the patient’s city of birth in the Basilicata region. We speculated about the impact of the expected amino acid change p.(Val453Met) on the structure of the enzyme using several in silico analysis tools, all of which agreed on its deleterious effect.

## 2. Materials and Methods

### 2.1. Patient

A 41-year-old woman coming from the Basilicata region (Italy) was admitted to our hospital Fondazione Policlinico Universitario “Agostino Gemelli” IRCCS of Rome (Italy) to undergo *G6PD* enzymatic and genetic analyses due to prenatal investigations and before artificial insemination treatment. The patient did not report any previous event suggestive of *G6PD* deficiency. The patient reported a positive family history of favism occurring in two first cousins, one of paternal origin and one of maternal origin. The patient underwent a standard *G6PD* assay [11]. The dosage of the erythrocytic *G6PD* enzyme was chosen to evaluate the kinetic determination of G6PDH in erythrocytes, using the *G6PD* mensuration reagent kit with the Atellica CH 930 instrument (Siemens Healthcare GmbH, Erlangen, Germany). The enzyme dosage was expressed in U/g Hb, with a reference range of 9.2–13.8 U/g Hb. The analysis was extended to family members who signed the consent form.

### 2.2. Genomic Analysis

Genomic DNA was extracted from whole-blood samples using the QIAamp DNA Mini kit with the Qiacube instrument (Qiagen, Milan, Italy), following the manufacturer’s instructions. Quantitation of the extracted DNA was performed using the Qubit dsDNA BR fluorimetric assays (Life Technologies, Gaithersburg, MD, USA). The *G6PD* genotyping strategy routinely adopted in our laboratory consists of a “first-level” evaluation of the most common mutations occurring in the Mediterranean area, as reported in [12]. In the absence of mutation and in the presence of *G6PD* deficiency and/or a suggestive history of favism or G6PD-related disorders, a “second-level” analysis is carried out with full-gene Sanger sequencing.

The “first-level” molecular analysis was carried out using *RFLP* techniques, with fragment evaluations performed on agarose gels and via capillary electrophoresis, or targeted Sanger sequencing. In detail, for *G6PD* A-, *G6PD Cassano*, and *G6PD* Seattle PVs genotyping, we used PCR coupled with *RFLP* as the methodological approach. In contrast, PCR and Sanger sequencing were used for the *G6PD* Chatham variant. Additionally, since the *G6PD Mediterranean* variant is the most frequent among patients presenting to our institution, it was more strategic to analyse it directly with the Sanger sequencing method. Primers used for PCR amplification and Sanger sequencing were previously reported in [12].

Sanger sequencing was carried out for the *G6PD Chatam* and *G6PD Mediterranean* variants and for confirmatory screening following a positive or equivocal *RFLP* result. Sequencing reactions were ran on the 3500 Genetic Analyzer (Applied Biosystems, Foster City, CA, USA) and analysed using SeqScape™ software 3 (Applied Biosystems) using the reference sequence NM_001360016.2.

For the remaining *G6PD* common mutations, the *RFLP* protocol was carried out after the first PCR, with minor changes, as reported in [13]. The digested PCR tests were analysed on agarose gel or by using the capillary electrophoretic system Tape Station 4200 (Agilent Technologies, Santa Clara, CA, USA) with the High Sensitivity D1000 ScreenTape analysis (Agilent Technologies, Santa Clara, CA, USA), and the reagents were combined as recommended by the manufacturer.

### 2.3. In Silico Analysis

The potential effect of the novel *G6PD* variant on enzyme structure/function and the prediction of pathogenicity were evaluated by combining different META in silico tools from Varsome [14]. The META predictors established pathogenicity interpretation based on the combined evidence from multiple other in silico tools. In particular, BayesDel (addAF and noAF) is a pathogenicity scoring platform working with coding and non-coding SNVs and small indels [15]; MetaLR is an ensemble score integrating 9 prediction scores (SIFT, PolyPhen-2, GERP++, MutationTaster, Mutation Assessor, FATHMM, LRT, SiPhy, and PhyloP) and allele frequencies in a 1KG database [16]; MetaRNN is a recurrent neural network-based ensemble prediction score integrating 16 prediction scores (SIFT, Polyphen2_HDIV, Polyphen2_HVAR, MutationAssessor, PROVEAN, VEST4, M-CAP, REVEL, MutPred, MVP, PrimateAI, DEOGEN2, CADD, fathmm-XF, Eigen, and GenoCanyon), 8 conservation scores (GERP, phyloP100way_vertebrate, phyloP30way_mammalian, phyloP17way_primate, phastCons100way_vertebrate, phastCons30way_mammalian, phastCons17way_primate, and SiPhy), and allele frequency information from the 1000 Genomes Project (1000GP), ExAC, and gnomAD [17]; MetaSVM is an ensemble score using Support Vector Machines to integrate 9 prediction scores (SIFT, PolyPhen-2, GERP++, MutationTaster, Mutation Assessor, FATHMM, LRT, SiPhy, and PhyloP) and allele frequencies in a 1KG database [18]; REVEL is an ensemble method for predicting the pathogenicity of missense variants that integrates 10 prediction scores (MutPred, FATHMM v2.3, VEST 3.0, PolyPhen-2, SIFT, PROVEAN, MutationAssessor, MutationTaster, LRT, GERP++, SiPhy, phyloP, and phastCons) [16].

## 3. Results

The results obtained from the evaluation of *G6PD* enzymatic activity and the haematological profile of the patient are described in Table 1. The patient showed normal, although very close to the lower range, *G6PD* activity (9.2 U/g Hb; 80% enzymatic activity) and displayed an asymptomatic status.

From the genetic analysis of G6PD, a wild-type status for the *G6PD* Chatham and *G6PD Mediterranean* variants analysed by direct sequencing emerged. The same result was obtained using the *RFLP* technique regarding the *G6PD A-* and *G6PD Seattle* variants. From the *RFLP* enzymatic digestion of exon 11, associated with the *G6PD Cassano* variant (c.1347G>C, p.(Gln449His)), a positive restriction pattern suggestive of the presence of the variant emerged. In particular, the observed restriction pattern showed the presence of three different bands with bp sizes close to the typical *RFLP* pattern of *G6PD Cassano*. Consequently, the presence of the *G6PD Cassano* variant was initially hypothesized. The latter positive result obtained by *RFLP*, as per practice in our laboratory, was then reconfirmed by Sanger sequencing testing. Unexpectedly, the direct sequencing analysis showed the presence of the heterozygous variant c.1357G>A (NM_001360016.2). The variant is not found in the databases of the Exome Aggregation Consortium (ExAC), 1000 Genomes http://www.internationalgenome.org/, (accessed on 20 August 2024), the Single Nucleotide Polymorphism Database (dbSNP), ClinVar http://www.ncbi.nlm.nih.gov/clinvar/, (accessed on 20 August 2024), and LOVD https://www.lovd.nl/, ( accessed on 20 August 2024). For these reasons, the variant was considered novel.

**Figure 1:**
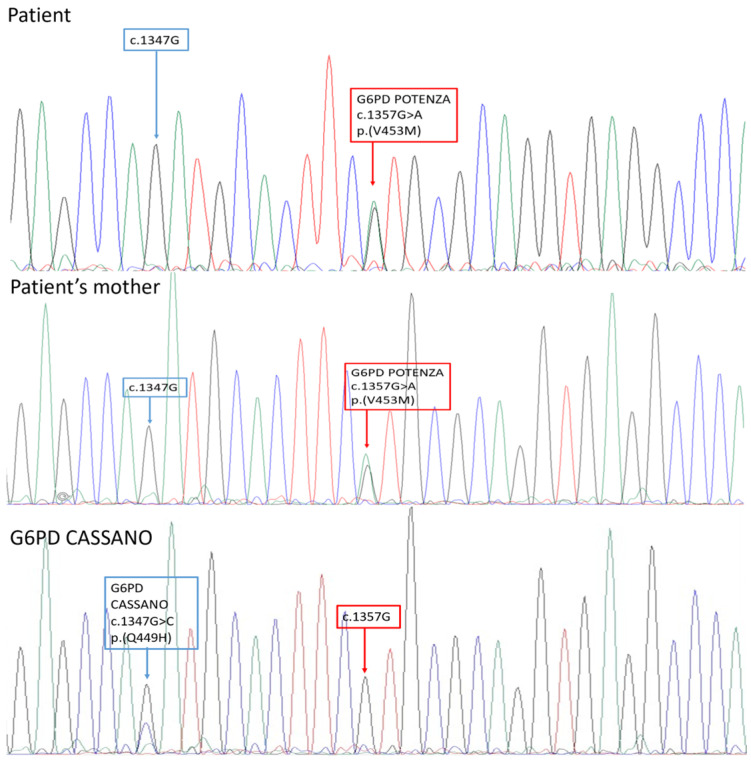
The sequencing results of the *G6PD* gene analysis. The blue arrows indicate the position of the *G6PD Cassano*, instead the red arrows indicate the position of the novel nucleotide change identified in this study. The patient resulted as a heterozygote for the c.1357 G>A variant in the *G6PD* gene; her mother was a heterozygote for the same variant. The figure demonstrates the proximity of the new variant to *G6PD Cassano* in a heterozygous patient shown as an example.

**Figure 2:**
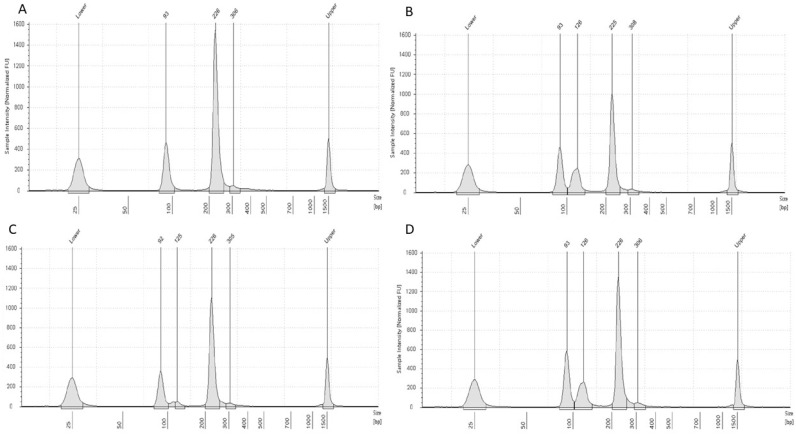
Capillary electrophoresis analysis of the enzymatic digestions of *Potenza* and *Cassano* PCR products. The figure shows the results obtained from the tape station capillary electrophoresis of the enzymatic digestions, performed with samples of the wild-type control (panel **A**), the patient (**B**), heterozygous *G6PD Cassano* (panel **C**), and the patient’s mother (panel **D**). Clear different profiles emerged from the wild-type and *Potenza* or *Cassano* analyses. However, the presence of two independent fragment patterns belonging to the presence of *Potenza* and *Cassano* variants is not evident. The altered pattern was also identified in the patient’s mother’s sample (panel **D**).

To fully characterized the familiar segregation of the novel variant, *G6PD* screening was offered to the patient’s mother, father, and brother (Figure 1 and Figure 2).

We speculated about the impact of the novel p.(Val453Met) amino acidic change on enzyme structure using several META in silico analysis tools from Varsome, accessed in September 2024, that are overall in agreement regarding its deleterious effect (Table 2) [14,19].

## 4. Discussion

**Figure 3:**
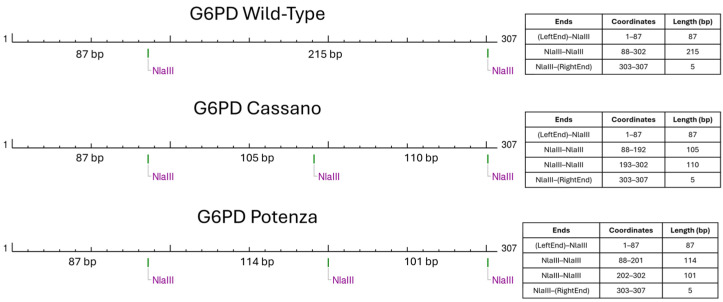
Schematic representation of restriction sites of NlaIII enzyme, obtained with NEBcutter V2.0.

To the best of our knowledge, the c.1357G>A variant was not previously reported in the main mutational databases and in the literature. It was decided to name the new variant “*G6PD Potenza*”, with regard to the patient’s city of birth in the Basilicata region. The molecular basis of such an unexpected *RFLP* result consisted of a peculiar restriction pattern created by the novel variant (Figure 3).

In fact, the presence of the *G6PD* c.1357G>A variant generated restriction sites and fragments with lengths that could be easily overlapped if analysed with electrophoretic approaches. This observation underlines the key role of the Sanger sequencing method, which still represents the gold-standard confirmatory approach.

**Figure 4:**
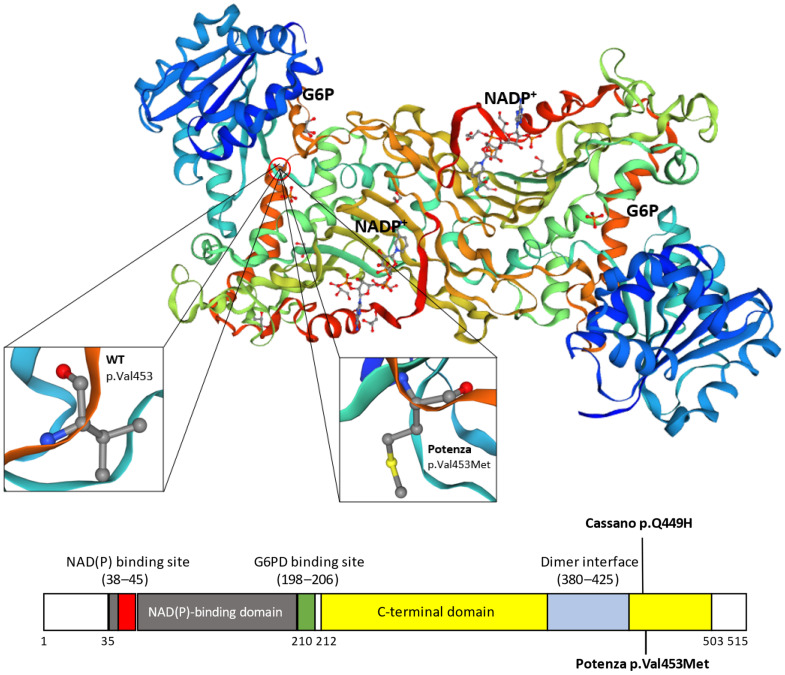
A schematic representation of the alteration in the *G6PD* protein functional domains. The variants *Cassano* (c.1347G>C p.Q449H) and *Potenza* (c.1357G>A p.Val453Met) are highlighted in black in the protein domains. The crystallographic structure of the human *G6PD* enzyme, assembled from https://swissmodel.expasy.org, (accessed on 10 August 2024).

The p.(Val453Met) substitution is located in a region of the *G6PD* enzyme that is important for maintaining its structural stability and catalytic function. Valine at position 453 is highly conserved across species, suggesting that it plays a critical role in enzyme function. The replacement by a methionine residue could potentially disrupt the enzyme’s tertiary structure or affect its binding affinity to substrates and cofactors [20] (Figure 4).

The average incidence of *G6PD* deficiency is 0.4% in the middle of Italy and 1% in Sicily, while it reaches an average value of 14.3% in Sardinia, with a peak of 25.8% in the province of Cagliari.

Rare new variants associated with a loco-regional Italian distribution were previously published by our group, suggesting the possible underestimation of the frequency of such novel *G6PD* variants [21,22]. In 1971, Yoshida and colleagues suggested a five-category classification system of *G6PD* mutations based on the residual enzymatic activity and the severity of the anaemia [6]. From the five classes, variants of classes IV and V (the latter was based on a single clinical case) have no clinical manifestations, unlike those of classes I-III. Specifically, the rare class I variants are defined as chronically associated with non-spherocytic haemolytic anaemia (CNSHA), while the polymorphic variants with a higher prevalence associated with the development of AHA (acute haemolytic anaemia) triggered by medicines, broad beans, or infection are assigned to classes II and III. Novel *G6PD* variants can be classified mainly according to the enzymatic activity in one or a few samples [13]. The classification of the variants adopted by the WHO takes into account the haematological consequences of *G6PD* deficiency and its degree of deficiency. In particular, the degree of *G6PD* deficiency is classified as class II if the residual activity is less than 10% and class III if it is greater than 10% [11]. Following a technical consultation held in January 2022, a revision (approved with minor changes by the WHO Malaria Policy Advisory Group) of the *G6PD* variant classification was proposed. This proposal was recently published in the WHO Bulletin [7]. Because CNSHA is a clinically distinct phenotype, all rare variants that cause CNSHA (previously classified as class I) are now classified as class A. In this revision, the previous class II and III were merged into class B. It is important to underline that class B *G6PD* variants could be considered a triggering factor of a pathological manifestation instead of a disease per se [11]. Based on the overall data highlighted in this case report, we could put forward a hypothesis for the classification of the new c.1357G>A, p.(Val453Met) variant as class B, and formerly class III, given the absence of pathological phenotypic or clinical manifestations and the enzymatic activity of 80%.

## 5. Conclusions

In this study, we describe the occurrence of the novel *G6PD* variant c.1357G>A, p.(Val453Met) in an Italian woman without any clinical symptoms. This case also highlights how the Sanger sequencing technique still represents an indispensable standard method for the final confirmation of germline variants that could be misinterpreted by using rapid approaches such as the *RFLP* technique. The availability of next-generation sequencing techniques as a high-throughput and effective strategy to perform germline mutational analysis could also be taken into account. The main limitation of this study is the lack of functional assays measuring the activity of the *G6PD* p.(Val453Met) enzyme compared to the wild type, which are considered crucial for determining the variant’s impact. The observation of a significant reduction in enzyme activity in vitro would support the hypothesis that the variant could be damaging [20].

Our study confirms the high heterogeneity of the distribution of *G6PD* variants in Italy, confirming the possibility of identifying novel, rare, or local *G6PD* variants in specific regions.

## Figures and Tables

**Table 1:** The haematological profile and *G6PD* enzymatic activity of the patient. Abbreviations: ALT, alanine aminotransferase; AST, glutamic oxaloacetylase; BU, unconjugated bilirubin; HGB, haemoglobin; MCH, mean haemoglobin content; MCHC, mean corpuscular haemoglobin concentration; MCV, mean cell volume; RBC, red blood cell; TBIL, total bilirubin.

Markers	Patient	Normal Range
RBC (×10^12^/L)	4.41	4.5~5.5
HGB (g/dL)	13.9	12.0~15.0
HCT (%)	40.4	36~46
MCV (dL)	91.5	83~101
MCH (pg)	31.6	27~32
MCHC (g/dL)	34.5	31.5~34.5
TBIL (mg/dL)	0.8	0.3~1.2
BU (mg/dL)	0.2	<0.3
ALT (UI/L)	17	<49
AST (UI/L)	18	<34
G6PDH/HGB (U/gHb)	9.2	9.2~13.8

**Table 2:** In silico prediction of the pathogenicity of the novel *G6PD* variant. For each tool, the computed score and the prediction of pathogenicity are reported (according to the reference range of pathogenicity).

Tool	Score	Score Range (Range for Damaging)	Prediction
BayesDel addAF	0.4643	−1.11707–0.750927	Pathogenic strong
MetaLR	0.9917	0–1	Pathogenic strong
MetaRNN	0.9624	0–1	Pathogenic strong
BayesDel noAF	0.4291	−1.31914–0.840878	Pathogenic moderate
MetaSVM	0.9432	−2–3	Pathogenic moderate
REVEL	0.835	0–1	Pathogenic moderate

## Data Availability

The data supporting this study’s findings are included in the manuscript.
